# Monotonic and Fatigue Strength of 3D-Printed Partially Stabilized Zirconia for Monolithic Restorations

**DOI:** 10.1016/j.identj.2026.109529

**Published:** 2026-03-28

**Authors:** Yuqing Lu, Li Wang, João Paulo Mendes Tribst, János Kodolányi, Cornelis J. Kleverlaan, Albert J. Feilzer, Amanda Maria de Oliveira Dal Piva

**Affiliations:** a Department of Dental Materials Science, Academic Centre for Dentistry Amsterdam (ACTA), Universiteit van Amsterdam and Vrije Universiteit, Amsterdam, The Netherlands; b Department of Reconstructive Oral Care, Academic Centre for Dentistry Amsterdam (ACTA), Universiteit van Amsterdam and Vrije Universiteit, Amsterdam, The Netherlands; c Jiangsu Key Laboratory of Advanced Food Manufacturing Equipment and Technology, School of Mechanical Engineering, Jiangnan University, Wuxi, Jiangsu, China

**Keywords:** 3D printing, Additive manufacturing, Zirconia, Flexural strength, Fatigue, Surface finishing

## Abstract

**Objectives:**

To investigate the monotonic and fatigue strength of 3D-printed 5 mol% yttria partially stabilized zirconia (5Y-PSZ) and the influences of printing layer orientation and finishing protocol.

**Methods:**

Bar-shaped 5Y-PSZ specimens (1.0 mm x 1.0 mm x 12.0 mm) were 3D-printed via stereolithography, followed by debinding and sintering. The specimens were randomly divided into 2 groups according to printing layer orientations: parallel or perpendicular to the tensile surface of a 3-point bending test. The specimens of each printing layer orientation were subsequently submitted to different surface finishing protocols: as-sintered, polished, and glazed. The monotonic strength of each group was determined using a ball-in-hole device. A step-wise fatigue test was conducted for the polished and glazed specimens.

**Results:**

For monotonic strength, the parallel specimens (640 ± 99 MPa) were stronger than the perpendicular (507 ± 48 MPa); polishing generated higher strength for both parallel (782 ± 134 MPa) and perpendicular (645 ± 160 MPa) orientations; while the effect of glazing was not significant, the glazed perpendicular specimen (623 ± 97 MPa) presented similar strength to the glazed parallel specimens (581 ± 117 MPa). However, the fatigue strength was similar in the evaluated groups (parallel and polished: 418 ± 115 MPa; perpendicular and polished: 404 ± 55 MPa; parallel and glazed: 406 ± 41 MPa; perpendicular and glazed: 371 ± 86 MPa).

**Conclusion:**

Both printing layer orientation and surface finishing influenced the monotonic strength of 3D-printed 5Y-PSZ, while fatigue strength did not differ significantly among polished and glazed specimens with different orientations.

## Introduction

Zirconia ceramics play a crucial role in restorative dentistry.^1^ The first generation of zirconia, 3 mol% yttria–stabilized zirconia polycrystals (3Y-TZP), is well-known for its high flexural strength, while its high opacity, limits it to be used only as substrate for load-bearing areas. Translucency was later improved by reducing alumina content in the ceramic powder, though this enhancement remains modest.^2^ Subsequently, incorporating 4–6 mol% of yttria enhances the cubic phase content in sintered zirconia,^1^ significantly improving translucency. This advancement enables the use of zirconia ceramics for monolithic restorations, even in anterior regions. Although this composition reduces mechanical properties, zirconia still outperforms other esthetic ceramic materials in terms of durability.^3^

Conventional manufacturing of zirconia restorations involves subtractive manufacturing, referring to milling the shape from a partially or fully sintered commercial blank. In recent years, an emerging technique of additive manufacturing, also known as 3D printing, has gained considerable interest as an alternative for ceramic restorations due to its significantly lower material waste, higher shaping capabilities and the possibility of massive production.^3^, ^4^, ^5^, ^6^ For dental applications, the current dominating technique is vat photopolymerization,^3^^,^^4^^,^^7^ specifically digital light processing (DLP) and stereolithography (SLA). This type of technique involves the use of a photosensitive monomer resin loaded with ceramic powder, which is selectively cured in a vat, followed by debinding and sintering to remove the polymer and fully densify the ceramic.^8^^,^^9^ A recent study has shown that the 3D-printed monolithic crowns have trueness and margin quality comparable to the milled,^10^ which demonstrates its promising applications. Among the printable ceramic materials, 3Y-TZP is the most highly developed, with mechanical properties comparable to commercial zirconia for milling.^11^, ^12^, ^13^, ^14^, ^15^ However, monolithic zirconia such as the 5 mol% yttria-partially stabilized zirconia (5Y-PSZ) is relatively limited.

The mechanical properties of 3D-printed materials are dependent on the orientation of printing layers, as is the case for ceramics printing techniques that achieve full densification not during printing but by subsequent thermal treatments.^16^^,^^17^ Previous studies have found that such different printing layer orientations resulted in different monotonic and fatigue strengths of 3Y-TZP, which are related to not only quality of interlayer bonding but also surface defects and roughness.^18^, ^19^, ^20^ Therefore, a good surface quality is significant for the strength of 3D-printed ceramics by reducing stress concentration at the surface. Additionally, for clinical restorations, an appropriate surface finishing is essential for esthetical purposes and reduction of biofilm formation, bacterial adhesion, and tooth wear.^21^^,^^22^ To achieve that, polishing and glazing are the most used finishing techniques. However, the impact of various finishing techniques on the fatigue behavior of 3D-printed monolithic zirconia remains poorly understood, as does the extent to which the influence of printing layer orientations can be minimized or eliminated.

When investigating the fracture behaviour of dental ceramics, flexural strength is commonly used to describe their ability to withstand a single occlusal load without immediate failure. In this study, the monotonic strength of 5Y-PSZ by SLA 3D printing, as well as the effect of printing layer orientation and finishing protocols were evaluated using a 3-point bending test. In the oral environment, dental ceramics are subjected to functional masticatory forces that act as cyclic loading. Such cyclic loading can promote subcritical crack growth, leading to failure at stresses below the material’s nominal strength. Therefore, the fatigue behavior of 3D-printed 5Y-PSZ was also investigated and compared between polished and glazed surface conditions.

## Materials and methods

### Specimen’s preparation

**Fig. 1:**
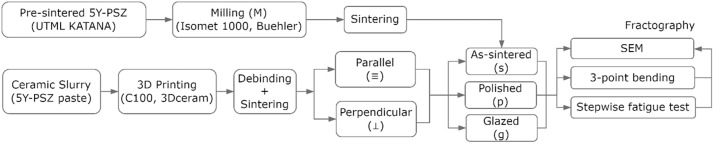
Flowchart of the study design.

The study’s design is presented in Figure 1. A 3D model of the bar-shaped specimen (1.0 mm x 1.0 mm x 12.0 mm^18^^,^^19^^,^^23^, ^24^, ^25^) was created in Standard Tessellation Language (STL). Before manufacturing, the 3D model was enlarged based on shrinkages according to different axes during printing (20% in the X–Y plane and 22% in the Z direction). The adjusted 3D files were exported to an SLA printing machine (C900; 3Dceram, France) to produce 150 specimens. For that, a paste-like zirconia slurry was prepared using 5Y-PSZ powder dispersed in a homogeneous mixture of photocurable monomers with a solid loading of 58 vol%. The zirconia powder consisted of zirconium oxide (> 90.0 wt%), yttrium oxide (8.8 wt%) and other remaining components (Al_2_O_3_, SiO_2_, Fe_2_O_3_, TiO_2_) (<0.1 wt%). The low-viscosity photosensitive monomers included trimethylolpropane triacrylate (TMPTA) and 1,6-hexanediol diacrylate (HDDA) with diphenyl (2,4,6-trimethyl benzoyl) phosphine oxide (TPO) monomer applied as the photoinitiator. To reduce the viscosity of the slurry, a dispersant (alkylolammonium salt of a copolymer with acidic groups) was added. A printing layer thickness of 25 μm was used under a light intensity of 90 mW/cm^2^. After printing, the specimens were removed from the platform and cleaned with isopropyl alcohol. Then, the specimens underwent a debinding process up to 600°C with a slow heating rate of 0.2°C/min, with dwell times of 2 h at 450°C and 600°C.^26^ The specimens were subsequently sintered at 1520°C for 4h.

For comparison, a group of milled specimens was obtained from commercial monotonic zirconia (KATANA UTML; Kuraray, Tokyo, Japan), which includes 87-92 wt% zirconium oxide, 8-11 wt% yttrium oxide and 0-2 wt% other oxides and pigments.^27^ A diamond-coated saw in a precision cutting machine (Isomet 1000; Buehler, Lake Bluff, IL) was used to cut 15 bar-shaped specimens for the monotonic test. Then, the specimens were sintered in a furnace at 1500°C for 2h, as recommended by the manufacturer. The final dimensions of each specimen (1.0 × 1.0 × 12.0 mm) were inspected using a digital caliper (Absolute 500-196-20, Mitutoyo, Takatsu-ku, Japan) prior to conducting the mechanical tests.

The 3D-printed specimens were randomly divided into 2 printing layer orientations according to the positioning of printing layers: parallel (≡) or perpendicular (⊥) to the tensile surface in the following bending test, as illustrated in the previous study.^19^ The 3D-printed specimens were submitted to 3 different surface protocols: as-sintered (s), polished (p), or glazed (g). For polishing, the specimens were wet-grounded using SiC abrasive papers (Ecomet; Buehler Ltd., Evanston, IL) with final grit sizes of P2000. The glaze layer was applied using a glaze paste (Cerabien FC Paste Stain Glaze; Kuraray Noritake, Tokyo, Japan) followed by the manufacturer-recommended firing procedure, which has a heating rate of 45°C/min, a highest temperature of 750°C and a holding time of 1 min.

### Monotonic strength test

Fifteen specimens (n = 15) from the milled and 3D-printed specimens (Ms, ≡s, ⊥s,≡p, ⊥p, ≡g and ⊥g) were used to determine monotonic strength (3-point bending) using a universal testing machine (Instron 6022; Instron Limited, High Wycombe, UK) with a ball-in-hole device.^18^^,^^19^^,^^23^, ^24^, ^25^ This device is composed of a metal base with a hole (10.1 mm diameter) and a stainless steel ball (10 mm diameter). The specimens were placed in the inner space of the metal base, which matched the length and width of the specimens with 2 supports 10 mm apart. The load was applied in the middle of the specimen surface using a load cell (100 N) at a crosshead speed of 0.5 mm/min. The monotonic strength (in MPa) was calculated according to the following equation:

*σ*=3*Pl*/(2*bh*^2^)

Where P is the maximum load, l is the distance between the 22 supports, b represents the specimen width, and h is the specimen thickness. The monotonic strength of all evaluated groups was analysed using one-way ANOVA and 2-parameter Weibull analysis. For the 3D-printed specimens, strength data were also submitted to 2-way analysis of variance (ANOVA) to evaluate the effects of printing layer orientation and surface condition. The statistical significance was set at a level of 0.05.

### Fatigue test

The fatigue behavior of the polished and glazed 3D-printed specimens (≡p, ≡g, ⊥p, ⊥g) was determined by a stepwise test (n=15), while the as-sintered specimens were not evaluated. The test was carried out using a fatigue machine (Fatigue Tester, ACTA, Netherlands) with the same ball-in-hole device and specimen dimension as the monotonic test.^23^ The stepwise protocol started from 5N for 10,000 cycles and increased by 5N for the next stress level until the specimen breaks, at a frequency of 3 Hz. The failure load and the number of cycles at the failure moment were recorded. To compare the fatigue strength and survival probability of all the groups, the data from the fatigue test were submitted to Kaplan-Meier and Wilcoxon tests.

### Scanning electron microscopy (SEM)

One representative specimen from each evaluated group was analysed using SEM (EVO LS15; Carl Zeiss) at 500x magnification to examine surface morphology. The fracture surfaces of 3D-printed specimens were collected and checked under optical microscopy to identify the location and type of their fracture origins. Fracture surfaces of 3D-printed specimens were collected and preliminarily inspected using optical microscopy. Representative specimens were selected and analysed under SEM at varying magnifications to identify the critical defect which initiated fracture. Prior to the SEM observations, the specimens were cleaned in an ultrasonic bath with 90% ethanol, and then gold-coated using an ion sputter coater in a low-pressure atmosphere.

## Results

**Fig. 2:**
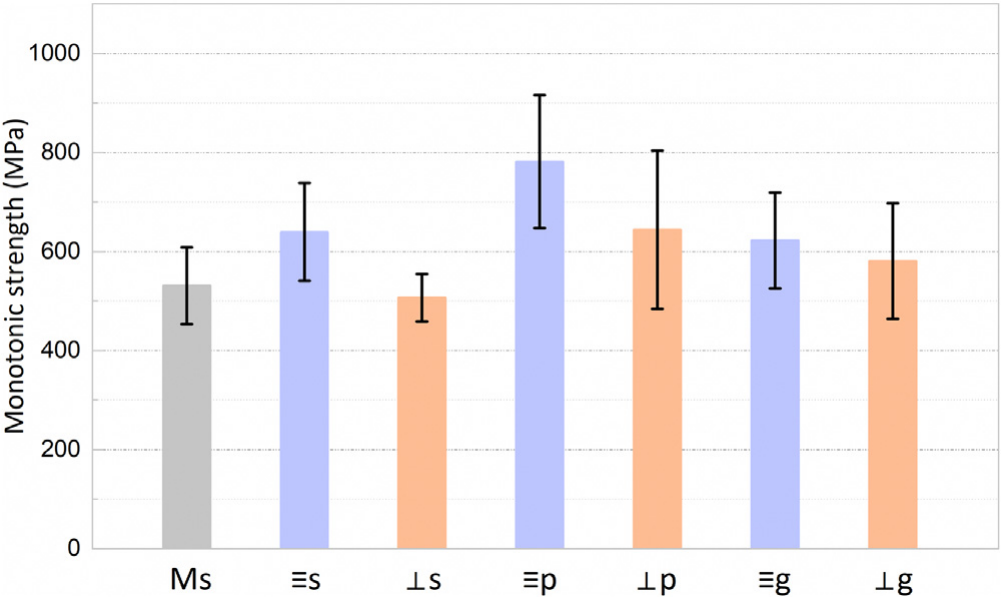
Mean values and standard deviations of monotonic strength of the milled as-sintered group (Ms) and the 3D-printed groups (≡s: parallel and as-sintered; ⊥s: perpendicular and as-sintered: ≡p: parallel and polished; ⊥p: perpendicular and polished; ≡g: parallel and glazed; ⊥g: perpendicular and glazed).

**Fig. 3:**
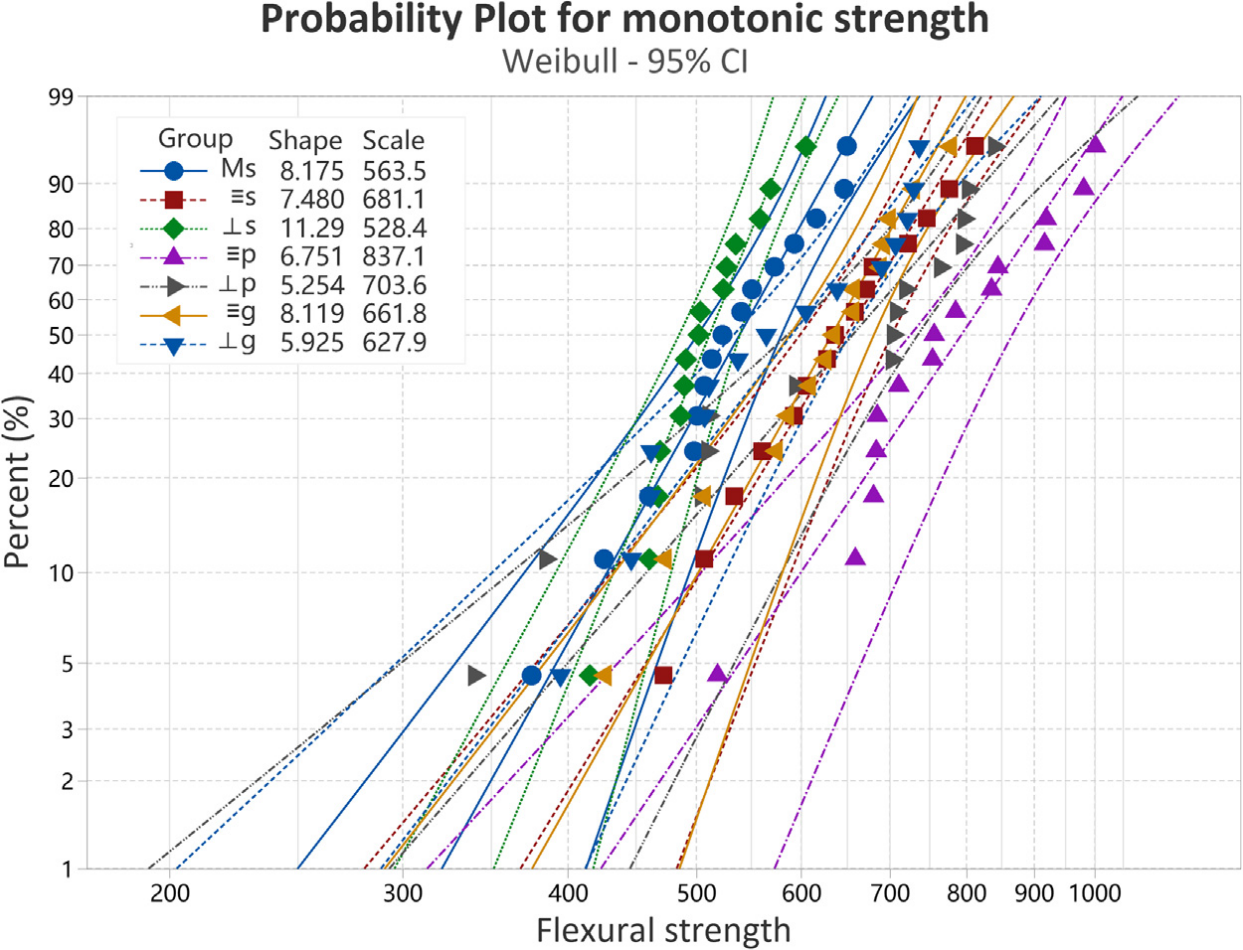
Weibull 95% confidence interval plots for the milled as-sintered group (Ms) and the 3D-printed groups (≡s: parallel and as-sintered; ⊥s: perpendicular and as-sintered: ≡p: parallel and polished; ⊥p: perpendicular and polished; ≡g: parallel and glazed; ⊥g: perpendicular and glazed).

The mean values and standard deviations of the monotonic strength are presented in Figure 2. One-way ANOVA showed a significant difference between all the evaluated groups (F = 10.715, *p* < .001). The milled specimens did not exhibit higher monotonic strength than the 3D-printed specimens. For the 3D-printed specimens, 2-way ANOVA revealed that Printing layer orientation (F = 18.628, *p* < .001) and Surface condition (F = 12.533, *p* < .001) influence the strength. However, the interaction of the factors Printing layer orientation*Surface condition was not found to have a significant effect (F = 1.665, *p* = .195). While the parallel specimens showed higher mean values than the perpendicular for the as-sintered and polished surface conditions, no significant difference was observed between the orientations for the glazed groups. Polishing enhanced the monotonic strength of both parallel and perpendicular specimens, whereas glazing did not demonstrate a significant effect. According to the probability plots (Figure 3), while the perpendicular specimens exhibited a higher reliability than the parallel for the as-sintered specimens, they displayed lower Weibull modulus for the polished or glazed surface conditions.

**Fig. 4:**
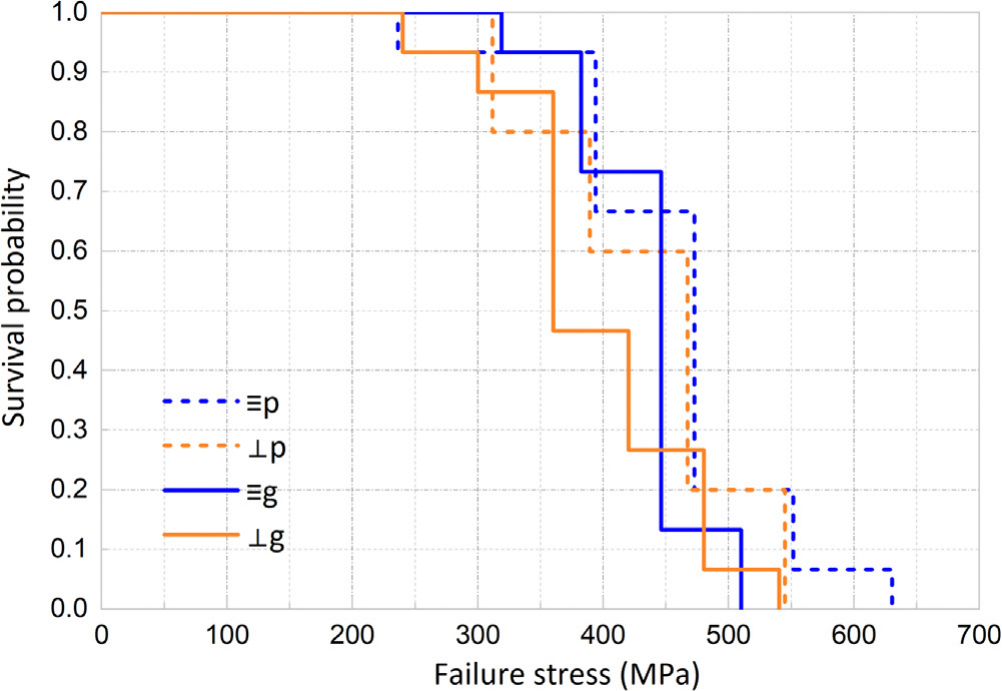
Survival plots of the polished and glazed 3D-printed specimens by Kaplan-Meier. (≡p: parallel and polished; ⊥p: perpendicular and polished; ≡g: parallel and glazed; ⊥g: perpendicular and glazed).

As illustrated in Figure 4, Kaplan-Meier analysis showed that the evaluated 3D-printed groups had similar survival curves, which is in accordance with the observation that no significant difference was identified in the mean fatigue strength, as presented in Table 1. Moreover, the survival probabilities and 95% confidence intervals at specific stress levels were presented in Table 1. At an applied stress level of 450 MPa, the parallel glazed specimens exhibited a lower survival probability than the parallel polished specimens. However, no significant differences were observed at other stress levels.

**Table 1:** Fatigue strength (MPa) and survival probabilities (%) at different stress.

Group	≡p	⊥p	≡g	⊥g
Fatigue strength (MPa) (95% CI)	452 (407 - 498)	424 (383 - 464)	436 (412 - 462)	408 (367 - 449)

Applied stress	Survival Probability (%) (95% CI)			

350 MPa	0.93 (0.81 – 1.00)	0.80 (0.60 – 1.00)	0.93 (0.81 - 1.00)	0.87 (0.69 – 1.00)
400 MPa	0.67 (0.43 - 0.91)	0.60 (0.35 - 0.85)	0.73 (0.50 - 0.96)	0.47 (0.21 - 0.72)
450 MPa	0.67 (0.43 - 0.91)	0.60 (0.35 - 0.85)	0.13 (0.00 - 0.31)	0.27 (0.04 - 0.49)
500 MPa	0.20 (0.00 - 0.40)	0.20 (0.00 - 0.40)	0.13 (0.00 - 0.31)	0.06 (0.00 - 0.19)

*≡p: parallel and polished; ⊥p: perpendicular and polished; ≡g: parallel and glazed; ⊥g: perpendicular and glazed.

**Fig. 5:**
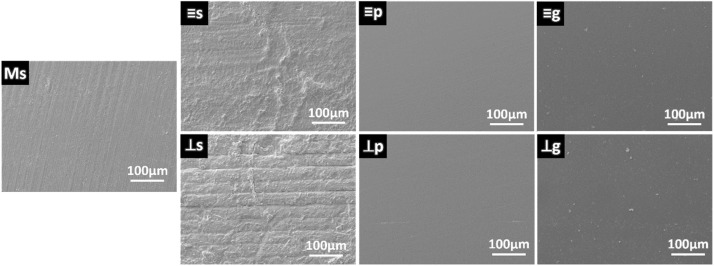
SEM images show the surface conditions of the milled as-sintered group (Ms) and the 3D-printed groups (≡s: parallel and as-sintered; ⊥s: perpendicular and as-sintered: ≡p: parallel and polished; ⊥p: perpendicular and polished; ≡g: parallel and glazed; ⊥g: perpendicular and glazed).

As presented in Figure 5, the SEM images revealed that milled zirconia exhibited a relatively smooth surface, although grooves resulting from the milling tool remained discernible. On the parallel 3D-printed specimens, surface scratches caused by the scraper of the 3D printing machine were observed along with a rough microstructure, which was attributed to the residual ceramic slurry. The perpendicular orientation exhibited a rougher surface with residual slurry and uniform grooves, which is known as the step effect associated with the layer-by-layer printing process. Polishing effectively produced a smooth surface on the 3D-printed specimens; however, some interlayer microcracks persisted in the perpendicular specimens. A similarly smooth surface condition was also achieved by applying a glaze layer to both orientations.

**Fig. 6:**
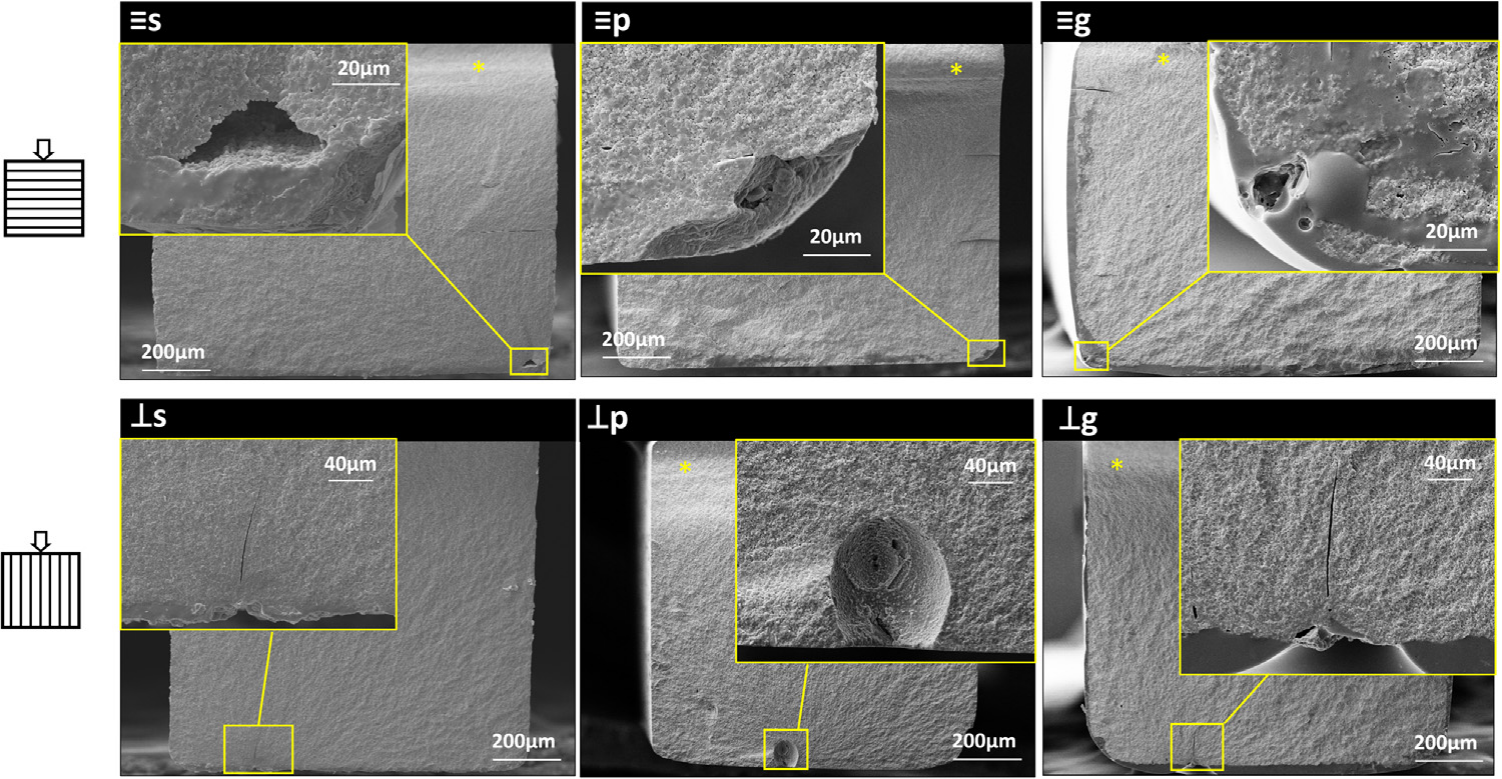
Typical fracture origins of the evaluated 3D-printed specimens. (≡s: parallel and as-sintered; ⊥s: perpendicular and as-sintered: ≡p: parallel and polished; ⊥p: perpendicular and polished; ≡g: parallel and glazed; ⊥g: perpendicular and glazed).

As presented in Figure 6, fractographic analysis revealed that fracture origins were predominantly located at or near the tensile surfaces and edges of the specimens. Both orientations shared similar types of critical defects, such as pores and microcracks. Consistent with prior findings in 3Y-TZP,^18^^,^^19^ these pores in 5Y-PSZ tended to have a long axis oriented parallel to the printing layers. Microcracks were identified on the lateral surfaces and aligned parallel to the printing layers. Additionally, in glazed specimens, bubbles within the glaze layer were also identified as potential fracture origins. No significant difference was observed in the defect type of fracture origins between the monotonic and fatigue tests.

## Discussion

In this study, the facture behavior of 3D-printed 5Y-PSZ and the influences of printing layer orientation and surface condition were investigated. The 3D-printed zirconia demonstrated monotonic strength comparable to that of commercial monolithic zirconia, with the parallel specimens exhibiting even higher strength. Both printing layer orientation and finishing protocol affect the monotonic strength. The parallel orientation and polishing protocol tend to generate higher average strength. However, the evaluated printing layer orientations and surface finishing protocols did not generate a significant difference in fatigue strength. Therefore, both commonly-used finishing techniques including polishing and glazing are acceptable for 3D-printed ceramic restorations.

Through technical advancements in recent years, an increasing number of studies showed that the 3D-printed 3Y-TZP, especially through vat photopolymerization, including DLP and SLA, have achieved satisfying mechanical properties comparable to commercial blank by conventional methods such as cold isostatic pressing.^3^^,^^28^^,^^29^ On the contrary, only a few previous studies investigated the mechanical properties of 3D-printed dental 5Y-PSZ including 3 studies^16^^,^^30^^,^^31^ using DLP and 1 study^32^ using an extrusion-based technique of direct ink writing. The monotonic strength of the strongest group through SLA in this study was comparable to that by DLP, which ranges from 568 to 657 MPa.^16^^,^^30^^,^^31^ Those values were notably higher than that of direct ink writing, which may be attributed to the relatively lower powder concentration of the latter.

In this study, the conventional monolithic zirconia did not show a significant higher monotonic strength or reliability than the 3D-printed specimens regardless of printing layer orientations. It is important to note that the same sintering protocol was applied to both milled and printed specimens. However, the materials were in different states before sintering: pre-sintered in the case of milled zirconia and debinded in the case of printed zirconia. Therefore, the selected protocol may not represent the optimal sintering condition for the milled specimens. Also, the milled and 3D-printed specimens in this study were produced using different starting powders, which may have contributed to variations in particle size, chemical composition, yttrium content and repartition. Furthermore, the yttria content of the evaluated commercial zirconia blank, while classified as 5Y-PSZ in a previous study,^2^ was identified to contain a higher percentage of 5.9 mol% in another study.^33^ This discrepancy could result in a higher proportion of the cubic phase in the sintered material, affecting its mechanical properties.^34^ Additionally, pigments incorporated into the commercial zirconia ceramics could further degrade mechanical performance.^35^ Therefore, the milled zirconia included in this study served as a reference rather than as a direct comparison between manufacturing techniques. Future studies using identical raw materials and optimized processing conditions are necessary to establish a more conclusive comparison between conventional subtractive and additive manufacturing techniques.

The influence of printing layer orientation on mechanical properties has been widely discussed for 3D-printed materials without exception for ceramics. The previous studies^18^^,^^19^ on SLA 3D-printed 3Y-TZP found that this difference could be attributed to orientation-dependent surface roughness and the orientation of the critical defects such as elliptical pore and interlayer microcracks as the result of the manufacturing process. In this study, similar defects were found in SLA-manufactured 5Y-PSZ by fractography. Porosity is a common type of flaw in both 3D-printed and milled ceramics. In contrast, interlayer microcracks are typical defects of additively manufactured ceramics and may result from insufficient interlayer bonding or deformation of the green body.^18^^,^^19^ These microcracks may exist as small flaws prior to loading but can propagate and enlarge under mechanical stress, which can significantly compromise mechanical strength; therefore, efforts are demanded to prevent these defects during fabrication. In this study, the parallel specimens exhibited higher strength and reliability than the perpendicular specimens under both polished and glazed conditions, suggesting lower sensitivity to porosity and crack-related defects. While the parallel orientation exhibited 26% higher mean monotonic strength compared to the perpendicular orientation for the as-sintered condition, this percentage dropped into 21% for the polished specimens, aligned with the 19% strength difference observed in polished specimens reported in the other study on DLP-manufactured 5Y-PSZ.^16^ Moreover, no significant difference in monotonic strength of glazed specimens. Fatigue strength was similar for both orientations by either polishing or glazing. These results demonstrated that the surface defects play a significant role in the strength differences between printing orientations, and such effect can be decreased by appropriate surface finishing. However, the influence of printing orientation cannot be completely eliminated by polishing alone, since intrinsic structural features, such as interlayer defects, also play an important role. Therefore, in clinical applications, the effect of printing orientations should be carefully considered to achieve optimal mechanical performance. Taken together, given the high strength observed in the present study, 3D-printed monolithic restorations may represent a promising option for clinical use.

However, while polishing can significantly reduce surface roughness, the surface defects are difficult to be removed completely, especially for the relatively deep scratches or microcracks on the rough lateral surface with step effect. This could explain why the 2 orientations had significantly different monotonic strength by polishing. Glazing, as another commonly used technique, has the risk of weakening the material strength since the glaze layer itself is a brittle structure. This is because the glazing protocol incorporated pores in the glaze layer and become the stress concentrator during monotonic loading.^25^ Furthermore, the application of a glaze layer can also generate a week interface between the ceramic and glass.^24^ In this study, the glazing technique did not improve monotonic strength such as polishing, that eliminated the strength difference between the printing layer orientations by covering surface defects. No significant difference in fatigue strength was observed for both finishing techniques, indicating both protocols are comparable for clinical usage. This result suggested the significance of exploring more finishing techniques that can reduce/heal surface defects effectively and further improve the mechanical properties of 3D-printed ceramic restorations.

One limitation of this study is that only 2 printing layer orientations were investigated. Secondly, the current finding in this study is limited to the condition involving tensile stress. The impact of printing layer orientation under compressive stress may differ, although compressive stress alone is not the typical cause of failure for dental restorations. Furthermore, future study on standard specimens and specific restoration types is necessary to establish a comprehensive understanding of the influence of printing layer orientation in interaction with surface finishing. Another critical property for monolithic restorative ceramics is translucency. The optimization of optical properties in zirconia ceramics is usually accompanied by a compromise in strength, based on studies on conventional zirconia ceramics.^36^ This trade-off can become even more challenging for the 3D-printed ceramics, as it is more difficult to generate a structure as dense as the conventional pressing technique, and a minor increase of porosity and defects could affect the light transmission. Further studies are needed to evaluate and optimize the optical properties of 3D-printed monolithic zirconia.

## Conclusion

Within the limitations, we conclude that:

1. Both printing layer orientation and finishing protocol affect the monotonic strength of 3D-printed 5Y-PSZ. The parallel orientation and polishing protocol tend to generate higher average strength.
2. The evaluated printing layer orientations and surface finishing protocols did not generate a significant difference in fatigue strength.
3. Despite some differences, polishing and glazing are both acceptable finishing protocols for 3D-printed ceramic restorations.
4. This study also highlighted the risk of surface defects on the monotonic strength of ceramics by 3D printing and the significance of effective surface finishing techniques to guarantee the performance of 3D-printed restorations.

## Author contribution

All authors contributed to the study conception and design. Material preparation, data collection and analysis were performed by Y.L., L.W., J.K. and A.D.P.. The first draft of the manuscript was written by Y.L. and all authors commented on previous versions of the manuscript. All authors read and approved the final manuscript.

## Conflict of interest

None disclosed.

## References

[1] Cesar P.F., Miranda RB de P., Santos K.F., Scherrer S.S., Zhang Y. (2024). Recent advances in dental zirconia: 15 years of material and processing evolution. Dent Mater.

[2] Zhang Y., Lawn BR. (2018). Novel Zirconia Materials in Dentistry. J Dent Res.

[3] Al Hamad K.Q., Al-Rashdan B.A., Ayyad J.Q., Al Omrani L.M., Sharoh A.M., Al Nimri A.M. (2022). Additive Manufacturing of Dental Ceramics: A Systematic Review and Meta-Analysis. J Prosthodont.

[4] Galante R., Figueiredo-Pina C.G., Serro AP. (2019). Additive manufacturing of ceramics for dental applications: A review. Dent Mater.

[5] Della Bona A., Cantelli V., Britto V.T., Collares K.F., Stansbury J.W. (2021). 3D printing restorative materials using a stereolithographic technique: a systematic review. Dent Mater.

[6] Chen Z., Li Z., Li J., Liu C., Lao C., Fu Y. (2018). 3D printing of ceramics: A review. Journal of European Ceramic Society.

[7] Branco A.C., Colaço R., Figueiredo-Pina C.G., Serro AP. (2023). Recent Advances on 3D-Printed Zirconia-Based Dental Materials: A Review. Materials.

[8] Bose S., Akdogan E.K., Balla V.K., Ciliveri S., Colombo P., Franchin G. (2024). 3D printing of ceramics: Advantages, challenges, applications, and perspectives. J Am Ceram Soc.

[9] Zakeri S., Vippola M., Levänen E. (2020). A comprehensive review of the photopolymerization of ceramic resins used in stereolithography. Addit Manuf.

[10] Silva SEG da, Silva NR da, Santos JV do N., Moreira FG de G., Özcan M., Souza RO de A e (2024). Accuracy, adaptation and margin quality of monolithic zirconia crowns fabricated by 3D printing versus subtractive manufacturing technique: A systematic review and meta-analysis of in vitro studies. J Dent.

[11] Lu Y., Wang L., Dal Piva A.M.O., Tribst J.P.M., Čokić S.M., Zhang F. (2024). Effect of printing layer orientation and polishing on the fatigue strength of 3D-printed dental zirconia. Dent Mater.

[12] Mei Z., Lu Y., Lou Y., Yu P., Sun M., Tan X. (2021). Determination of Hardness and Fracture Toughness of Y-TZP Manufactured by Digital Light Processing through the Indentation Technique. Biomed Res Int.

[13] Lu Y., Wang L., Dal Piva A.M.O., Tribst J.P.M., Nedeljkovic I., Kleverlaan C.J. (2023). Influence of surface finishing and printing layer orientation on surface roughness and flexural strength of stereolithography-manufactured dental zirconia. J Mech Behav Biomed Mater.

[14] Lu Y., Mei Z., Zhang J., Gao S., Yang X., Dong B. (2020). Flexural strength and Weibull analysis of Y-TZP fabricated by stereolithographic additive manufacturing and subtractive manufacturing. J Eur Ceram Soc.

[15] Tan X., Lu Y., Gao J., Wang Z., Xie C., Yu H. (2022). Effect of high-speed sintering on the microstructure, mechanical properties and ageing resistance of stereolithographic additive-manufactured zirconia. Ceram Int.

[16] Marsico C., Øilo M., Kutsch J., Kauf M., Arola D. (2020). Vat polymerization-printed partially stabilized zirconia: Mechanical properties, reliability and structural defects. Addit Manuf.

[17] Miura S., Shinya A., Ishida Y., Fujisawa M. (2022). Mechanical and surface properties of additive manufactured zirconia under the different building directions. J Prosthodont Res.

[18] Lu Y., Wang L., Dal Piva A.M.O., Tribst J.P.M., Nedeljkovic I., Kleverlaan C.J. (2023). Influence of surface finishing and printing layer orientation on surface roughness and flexural strength of stereolithography-manufactured dental zirconia. J Mech Behav Biomed Mater.

[19] Lu Y., Wang L., Dal Piva A.M.O., Tribst J.P.M., Čokić S.M., Zhang F. (2024). Effect of printing layer orientation and polishing on the fatigue strength of 3D-printed dental zirconia. Dent Mater.

[20] Dal Piva AM de O., Tribst J.P.M., Venturini A.B., Anami L.C., Bonfante E.A., Bottino M.A. (2020). Survival probability of zirconia-reinforced lithium silicate ceramic: Effect of surface condition and fatigue test load profile. Dent Mater.

[21] Dal Piva A.M.O., Contreras L.P.C., Ribeiro F.C., Anami L.C., Camargo S.E.A., Jorge A.O.C. (2018). Monolithic ceramics: Effect of finishing techniques on surface properties, bacterial adhesion and cell viability. Oper Dent.

[22] Janyavula S., Lawson N., Cakir D., Beck P., Ramp L.C., Burgess JO. (2013). The wear of polished and glazed zirconia against enamel. J Prosthet Dent.

[23] Machry R.V., Dapieve K.S., Cadore-Rodrigues A.C., Werner A., de Jager N., Pereira G.K.R. (2022). Mechanical characterization of a multi-layered zirconia: Flexural strength, hardness, and fracture toughness of the different layers. J Mech Behav Biomed Mater.

[24] Lu Y., Dal Piva A.M.O., Nedeljkovic I., Tribst J.P.M., Feilzer A.J., Kleverlaan C.J. (2023). Effect of glazing technique and firing on surface roughness and flexural strength of an advanced lithium disilicate. Clin Oral Investig.

[25] Lu Y., Dal Piva A.M.O., Tribst J.P.M., Feilzer A.J., Kleverlaan C.J. (2023). Does glaze firing affect the strength of advanced lithium disilicate after simulated defects?. Clin Oral Investig.

[26] Wang L., Yao L., Tang W., Dou R. (2023). Effect of Fe2O3 doping on color and mechanical properties of dental 3Y-TZP ceramics fabricated by stereolithography-based additive manufacturing. Ceram Int.

[27] KATANA Zirconia UTML – Ultra translucent zirconium dioxide with color graduation n.d.https://www.bego.com/cad-cam-solutions/materials/ceramic/katana-zirconia-utml/(accessed November 28, 2024).

[28] Mei Z., Lu Y., Lou Y., Yu P., Sun M., Tan X. (2021). Determination of Hardness and Fracture Toughness of Y-TZP Manufactured by Digital Light Processing through the Indentation Technique. Biomed Res Int.

[29] Lu Y., Mei Z., Zhang J., Gao S., Yang X., Dong B. (2020). Flexural strength and Weibull analysis of Y-TZP fabricated by stereolithographic additive manufacturing and subtractive manufacturing. J Eur Ceram Soc.

[30] Jung J.M., Kim G.N., Koh Y.H., Kim HE. (2023). Manufacturing and Characterization of Dental Crowns Made of 5-mol% Yttria Stabilized Zirconia by Digital Light Processing. Materials.

[31] Mirt T., Kocjan A., Hofer A.K., Schwentenwein M., Ivekovi´civekovi´c A., Bermejo R. (2023). Effect of airborne particle abrasion and regeneration firing on the strength of 3D-printed 3Y and 5Y zirconia ceramics. Dent Mater.

[32] Teegen I.S., Schadte P., Wille S., Adelung R., Siebert L., Kern M. (2023). Comparison of properties and cost efficiency of zirconia processed by DIW printing, casting and CAD/CAM-milling. Dent Mater.

[33] Inokoshi M., Shimizubata M., Nozaki K., Takagaki T., Yoshihara K., Minakuchi S. (2021). Impact of sandblasting on the flexural strength of highly translucent zirconia. J Mech Behav Biomed Mater.

[34] Čokić S.M., Vleugels J., Van Meerbeek B, Van Oosterwyck H, Inokoshi M., Zhang F. (2022). Mechanical properties-translucency-microstructure relationships in commercial monolayer and multilayer monolithic zirconia ceramics. Dent Mater.

[35] Čokić S.M., Li M., Huang S., Vleugels J., Van Meerbeek B., Zhang F. (2024). Coloring multilayer zirconia may affect its optical and mechanical properties. J Dent Res.

[36] Zhang F., Reveron H., Spies B.C., Van Meerbeek B., Chevalier J. (2019). Trade-off between fracture resistance and translucency of zirconia and lithium-disilicate glass ceramics for monolithic restorations. Acta Biomater.

